# Inclusion of nonrandomized studies of interventions in systematic reviews of interventions: updated guidance from the Agency for Health Care Research and Quality Effective Health Care program

**DOI:** 10.1016/j.jclinepi.2022.08.015

**Published:** 2022-09-19

**Authors:** Ian J. Saldanha, Gaelen P. Adam, Lionel L. Bañez, Eric B. Bass, Elise Berliner, Beth Devine, Noah Hammarlund, Anjali Jain, Susan L. Norris, Andrea C. Skelly, Kelly Vander Ley, Zhen Wang, Timothy J. Wilt, Meera Viswanathan

**Affiliations:** a Center for Evidence Synthesis in Health, Department of Health Services, Policy, and Practice, Department of Epidemiology, Brown University School of Public Health, Providence, RI, USA; b Evidence-Based Practice Center Program, Center for Evidence and Practice Improvement, Agency for Healthcare Research and Quality, Rockville, MD, USA; c Department of Medicine, Johns Hopkins School of Medicine, Baltimore, MD, USA; d Cerner Enviza, Inc., Kansas City, MO, USA; e Comparative Health Outcomes, Policy, and Economics (CHOICE) Institute, University of Washington School of Pharmacy, Seattle, WA, USA; f Department of Health Services Research, Management, and Policy, University of Florida, Gainesville, FL, USA; g Oregon Health and Science University, Portland, OR, USA; h Pacific Northwest Evidence-Based Practice Center, Portland, OR, USA; i Aggregate Analytics, Inc., Fircrest, WA, USA; j Department of Medical and Clinical Informatics and Clinical Epidemiology, Oregon Health and Science University, Portland, OR, USA; k Robert D. and Patricia E. Kern Center for the Science of Health Care Delivery, Mayo Clinic, Rochester, MN, USA; l Minneapolis VA Center for Care Delivery and Outcomes Research, University of Minnesota Schools of Medicine and Public Health, Minneapolis, MN, USA; m RTI International, Research Triangle Park, NC, USA

**Keywords:** Systematic reviews, Nonrandomized studies, Study eligibility, Guidance, Interventions, Effectiveness

## Abstract

**Objectives:**

We developed guidance to inform decisions regarding the inclusion of nonrandomized studies of interventions (NRSIs) in systematic reviews (SRs) of the effects of interventions.

**Study Design and Setting:**

The guidance workgroup comprised SR experts and used an informal consensus generation method.

**Results:**

Instead of recommending NRSI inclusion only if randomized controlled trials (RCTs) are insufficient to address the SR key question, different topics may require different decisions regarding NRSI inclusion. We identified important considerations to inform such decisions from topic refinement through protocol development. During topic scoping and refinement, considerations were related to the clinical decisional dilemma, adequacy of RCTs to address the key questions, risk of bias in NRSIs, and the extent to which NRSIs are likely to complement RCTs. When NRSIs are included, during SR team formation, familiarity with topic-specific data sources and advanced analytic methods for NRSIs should be considered. During protocol development, the decision regarding NRSI inclusion or exclusion should be justified, and potential implications explained. When NRSIs are included, the protocol should describe the processes for synthesizing evidence from RCTs and NRSIs and determining the overall strength of evidence.

**Conclusion:**

We identified specific considerations for decisions regarding NRSI inclusion in SRs and highlight the importance of flexibility and transparency.

## 1. Introduction

Systematic reviews (SRs) of interventions can include randomized controlled trials (RCTs) and nonrandomized studies of interventions (NRSIs).

### 1.1. Strengths and limitations of randomized controlled trials

High-quality RCTs have traditionally been considered the ideal study design for examining the effects of interventions. Methods employed in high-quality RCTs, such as prospective registration, randomization, allocation concealment, masking (of participants, trial personnel, and outcome assessors), and reporting of deviations from the study protocol, help RCTs to avoid or minimize the impact of threats to validity, such as selection bias, confounding, and reporting biases [1–3]. However, RCTs may bear important limitations that may limit the applicability of their findings to end-user interests and may present other challenges. Specifically, RCTs sometimes may (1) be unethical to perform due to the absence of clinical equipoise or other reasons; (2) apply narrow participant eligibility criteria and/or tightly controlled implementation of interventions and comparators; (3) focus on intermediate (or surrogate) outcomes instead of clinically important or patient-important outcomes; (4) not be able to detect true differences among comparators for some outcomes due to small sample sizes and/or short follow-up durations; and (5) have to draw from a very small population of interest (e.g., for rare diseases).

### 1.2. Defining nonrandomized studies of interventions and their types

NRSIs are observational or experimental studies of the effectiveness and/or harms of interventions, in which participants are not randomized to intervention groups [3,4]. Unfortunately, there is no consensus on NRSI terminology and study categorization; different researchers may refer to the same design using different terminology [5,6]. We agree with other methodologists that systematic reviewers should use study methods rather than study design labels to differentiate among NRSI types [7]. When considering study methods to specify NRSI types, we suggest the following five characteristics, although other characteristics may also be helpful:

1. presence of a comparison group receiving a different intervention or not receiving an intervention (controlled vs. uncontrolled/single group);
2. experimental nature (experimental [i.e., investigator assigns group] vs. nonexperimental);
3. type of control group (historic control vs. concurrent control vs. none);
4. presence of follow-up over time (yes [i.e., longitudinal] vs. no [i.e., cross-sectional]); and
5. temporality, in the case of longitudinal studies (prospective vs. retrospective).

The full report of this guidance provides a summary, including strengths and weaknesses, of common types of NRSIs based on the above-listed aspects [4].

### 1.3. Threats to the internal validity of nonrandomized studies of interventions

Potential threats to the internal validity of NRSIs are important for deciding whether NRSIs should be included in an SR. Sources of bias that are unique to NRSIs occur before or at the start of the intervention; sources of bias that occur after the intervention starts may be akin to those in RCTs [3]. The following three unique categories of bias frequently threaten the validity of NRSIs.

#### 1.3.1. Selection bias

NRSIs may be subject to a high risk of selection bias if at study baseline some potentially eligible participants, or their follow-up time, were excluded from the treatment or comparator groups, and such exclusion may have led to a biased estimate of the treatment effect [2].

#### 1.3.2. Confounding

NRSIs may be subject to a high risk of confounding if the treatment and comparator groups were imbalanced in terms of factors that were causes of both the choice of treatment and the outcome. A confounder is a third variable that is associated with the treatment and is a cause of the outcome but is not in the causal pathway between the treatment and the outcome (i.e., is not a mediator) [8]. Confounders may be known or unknown; known confounders may be measured or unmeasured in a given study.

#### 1.3.3. Misclassification

Intervention status may be misclassified because of an error in measurement [8]. If data on the intervention status are collected when the outcome (or risk of the outcome) is known, differential misclassification of the intervention status may occur [8]. Covariates and outcomes may also be misclassified.

In addition to the above threats to validity, many of the limitations described in the previous section on RCTs may also apply to NRSIs.

### 1.4. Design and analytical approaches to address threats to validity of nonrandomized studies of interventions

Traditional approaches to address confounding, such as matching and multivariable regression analysis, may be sufficient to ameliorate threats to validity. However, these approaches rely on the assumption that the full set of confounders is known and validly measured. Our full report provides summaries, assumptions, and examples of various common, advanced analytic approaches that, when used appropriately and under certain assumptions, may increase the possibility of causal inference in NRSIs, such as propensity scoring, instrumental variables, regression discontinuity, and difference-in-difference approaches [4]. Advancements in the design and analysis of NRSIs allow NRSIs in some topic areas to have a much more prominent role in decision-making, and not just as ancillary evidence to RCTs [9,10].

Reviews and meta-epidemiological studies that have empirically compared RCTs and NRSIs have generally found that the conclusions from both bodies of evidence tend to agree [11–14]. On the other hand, other recent analyses have suggested a lack of agreement [15,16]. The Randomized Controlled Trials Duplicated Using Prospective Longitudinal Insurance Claims: Applying Techniques of Epidemiology (RCT DUPLICATE) initiative is a large systematic evaluation of the ability of NRSIs using routine clinical data to replicate RCTs [17]. This initiative aims to quantify the differences between results from the two types of studies, as well as the factors that may explain the differences. Results from the first 10 emulations, which focused on insurance claims data on cardiovascular outcomes of antidiabetic or antiplatelet medications, were mixed; 80% of the emulations achieved agreement in estimates [18]. Thus, preliminary results in this limited clinical area support the conclusion that the selection of active comparator therapies with similar indications and use patterns increases agreement between results of NRSIs and RCTs [18].

In an SR of interventions, if conclusions in RCTs and NRSIs agree, the inclusion of NRSIs may help lend credence to conclusions from RCTs and/or broaden the applicability of the conclusions. If they disagree, NRSI evidence contributes new evidence and/or may serve as a “necessary complement to RCTs”. [15].

### 1.5. Objective

This new guidance updates the 2010 Agency for Healthcare Research and Quality (AHRQ) Evidence-based Practice Center (EPC) methods guide for effectiveness and comparative effectiveness reviews chapter on selecting observational studies for comparing medical interventions [19,20]. The new guidance is intended to inform decisions regarding the inclusion of NRSIs in SRs of the benefits and/or harms of interventions that are intended to work at the level of the individual patient, the clinic, the health system, or the broader population. We focus herein on the considerations for making the decision regarding the inclusion of NRSIs.

## 2. Methods

To develop this new guidance, AHRQ convened a 20-member workgroup that comprised 13 members representing eight of nine current AHRQ-appointed EPCs (https://effectivehealthcare.ahrq.gov/about/epc), three AHRQ representatives, one independent consultant with expertise in SRs, and three representatives of the AHRQ-appointed Scientific Resource Center (https://effectivehealthcare.ahrq.gov/about/src). The group met remotely approximately twice a monthfor10monthsandusedaninformalmethodtogenerate consensus. Workgroup members drafted various sections of the guidance, and the workgroup solicited input from all current EPCs through discussions at a virtual meeting and two anonymous online surveys. A draft report was circulated to all current EPC Directors and AHRQ EPC division members for review and comment. The final report, on which this manuscript is based, addressed these comments [4].

## 3. Results

We identified nine considerations that should inform decisions regarding the inclusion of NRSIs in SRs of interventions. We also describe how systematic reviewers should plan for the inclusion (or exclusion) of SRs early in the process. Although not discussed herein, the full report of this guidance provides 10 additional considerations regarding how NRSIs should be handled during subsequent stages of the SR (if the decision is made to include NRSIs): seven while conducting the SR and three while reporting it [4]. This paper focuses on the nine considerations for making the decision regarding the inclusion of NRSIs.

### 3.1. Considerations

Different SR topics and key questions may require different decisions regarding the inclusion or exclusion of NRSIs. Table 1 lists nine considerations that should inform the decisions (four during topic scoping and refinement, one during SR team formation, and four during protocol development).

#### 3.1.1. Considerations during topic scoping and refinement

Consideration 1: What are the decisional dilemmas and key questions being addressed, and how will the end user(s) of the SR use the evidence to inform decision-making?

When considering whether to include NRSIs in an SR, it is important to assess the extent to which the specific research questions addressed in relevant NRSIs align with the SR’s key questions. This assessment requires explicit consideration of whether the decisional dilemmas, and hence the key questions concern efficacy, effectiveness, or harm. RCTs generally are designed to determine efficacy (i.e., under the ideal circumstances), while NRSIs that include comparator groups generally focus on effectiveness (i.e., under the usual circumstances of routine practice). Accordingly, systematic reviewers should evaluate the extent to which RCTs and NRSIs are likely to address these dilemmas. A related question is whether NRSIs are likely to fill gaps in the RCT evidence base.

The extent to which available RCTs and NRSIs, taken together, are likely to address the populations, interventions, comparators, outcomes, and settings (PICOS) of the key questions should also be considered. The assessment requires consideration of whether known available RCTs and NRSIs address a PICOS-defined key question directly or indirectly, as well as the potential bias from NRSIs. If RCTs address the key question directly and NRSIs address them only indirectly, it may be reasonable to exclude NRSIs from the SR. If both NRSIs and RCTs address the questions directly, it may be best to include NRSIs because they may provide additional evidence and/or context.

Research on a topic may evolve in a way that increases or decreases the value of NRSIs. Early in the evolution of evidence on a topic, when limited evidence from RCTs may be available, it may be difficult to justify excluding NRSIs. In such a scenario, identification and synthesis of relevant NRSIs, with an acknowledgment of their limitations, could help articulate the need for RCTs. As the evidence evolves and RCT evidence accumulates, systematic reviewers should consider whether and how NRSIs may have evolved (or not) to complement the evidence from the RCTs. The inclusion of NRSIs in SRs in this context could help (1) assess how the overall evidence applies to routine practice and (2) reveal the intervention’s long-term effects and/or harms. When the evidence matures to a stage where large seminal RCTs are available, NRSIs may be excluded if the evidence from NRSIs is unlikely to alter conclusions gleaned from RCTs. In general, these considerations are consistent with a strategy that assesses the overall strength of evidence based on the best set of studies (i.e., the “best evidence” approach) [21].

Consideration 2: Is it logical and likely for RCTs to have addressed the key questions adequately?

For this consideration, we recommend specific considerations for each aspect of PICOS, as follows:

##### 3.1.1.1. Population.

What are the condition and population of interest? Are they likely to be adequately represented in RCTs?

##### 3.1.1.2. Interventions and Comparators.

Are they new or established? Do considerable variations exist in how they are implemented in clinical practice? Are the full ranges of interventions and comparators of interest likely to be adequately represented in RCTs?

##### 3.1.1.3. Outcomes.

What are the primary outcomes of interest, including benefits and harms? Are there outcomes of interest (e.g., long-term effects, long-term harms) for which RCTs may not be suitable, available, or feasible? Particularly for harms, is a comparator group required to answer the key question? Some outcomes may be particularly susceptible to measurement bias and confounding, so NRSIs that focus exclusively on such outcomes may be excluded if they report results that are likely to be biased. For example, NRSIs, when compared with RCTs, have been shown to overestimate the benefits of treatments for pain [22]. In an EPC SR of treatments for acute and chronic pain [22], systematic reviewers focused on RCTs because of the susceptibility of NRSIs to confounding and bias for subjective outcomes, such as pain and function. However, the systematic reviewers included large NRSIs for assessment of rare serious adverse events.

##### 3.1.1.4. Settings.

What settings are of primary importance? Are they likely to be adequately represented in RCTs?

Consideration 3: How serious is the risk of bias in NRSIs that address the key questions likely to be?

Systematic reviewers should consider whether causal inference is needed to answer the key question. They should also consider the level of methodologic rigor that would be required of NRSIs to allow meaningful conclusions, meet the needs of the end-user, and comply with contemporary standards for SRs. High-risk of bias studies, whether randomized or not, may not provide useful evidence and may be misleading.

Consideration 4: To what extent are NRSIs and RCTs likely to complement each other?

Is randomization required to answer the key question, particularly regarding benefits? Taken together, would the body of evidence from RCTs cover diverse populations and/or implementations of the intervention?

#### 3.1.2. Consideration during systematic review team formation

Consideration 5: As noted in Table 1, when NRSIs are planned to be included, the SR team should include members who are familiar with topic-specific data source considerations and advanced analytic methods for NRSIs.

#### 3.1.3. Considerations during protocol development

As noted in Table 1

Consideration 6: Systematic reviewers should specify in the protocol the study design methods or features that will be eligible for the SR.

Consideration 7: The decision to include or exclude NRSIs should be noted and explained.

Consideration 8: Systematic reviewers should also discuss the potential implications of the decision to include or exclude NRSIs.

Consideration 9: If NRSIs are going to be included, the protocol should also describe the processes for synthesizing evidence from RCTs and NRSIs and determining the overall strength of evidence.

## 4. Discussion

### 4.1. Summary of guidance

The current guidance handles NRSI evidence as a potentially important source of information depending on the topic and related decisional dilemmas and acknowledges the limitations and potential benefits of doing so. The main change from the previous (2010) guidance is the overall approach to decisions about including NRSIs. Instead of recommending that NRSIs be included only if RCTs are insufficient to address the key question, or that NRSIs always be included, the current guidance considers NRSIs as potentially important.

The considerations provided in this guidance may not be an exhaustive list. Whatever decision is made regarding including NRSIs, it is crucial for systematic reviewers to be transparent in reporting the decision and justifying it in the SR protocol and the description of methods in the final publication. Reporting study findings in the context of their limitations (regardless of study design) remain of primary importance.

### 4.2. Challenges during guidance development

Our discussions and the drafting and revising of this report over many months, with subsequent input from various EPC directors, occurred in the context of an evolving climate regarding how the global SR community and, more broadly, the global intervention research community view studies in which participants are not randomized. Naturally, there were various points of view on this issue within and beyond the workgroup. We view the ability to achieve consensus despite varying viewpoints as a reflection of the strength of the process.

Relatively early in our deliberations, it became clear that a one-size-fits-all approach to this guidance was neither desired nor appropriate. The current guidance does not suggest that all SRs follow the same decision pathway. We decided to abandon the decisional framework (flow diagram) recommended in the 2010 guidance [19,20] because we believe that the considerations for decisions regarding the inclusion or exclusion of NRSIs in SRs are currently too complex and customized to be fully captured in a figure. Instead, we call for flexibility in the decision-making and describe important considerations that are intended to guide the decisions.

### 4.3. Implications and limitations

The guidance provided in this paper may have significant implications. Although it may improve the utility of the final SR product to end-users, it is likely to require more time, methodological expertise, and resources to complete the SR than if NRSIs were excluded. In addition to balancing these tradeoffs, a continuing and crucial concern pertains to the potential threats to the validity of NRSIs.

The guidance applies to SRs conducted in the EPC program and may also apply to other SRs. A potential limitation with this guidance is that some considerations, particularly those during topic scoping and refinement (e.g., the extent to which NRSIs and RCTs are likely to complement each other) may be challenging to assess early in an SR. This emphasizes the importance of including both clinical topical as well as methodologic experts on the SR team. Finally, we anticipate that, as is generally true, this guidance will need updating in the future.

## 5. Conclusion

Systematic reviewers of intervention effects must decide whether to restrict study inclusion to RCTs or to also include NRSIs. We identified considerations to inform such decisions. During topic scoping and refinement, considerations were related to the specific clinical decisional dilemma, the adequacy of RCTs to address the key questions, the risk of bias in NRSIs, and the extent to which NRSIs and RCTs may complement each other. Whatever decision is made regarding including NRSI inclusion, it is crucial for systematic reviewers to be transparent in explaining the decision in the SR protocol and the description of methods in the final publication.

## Figures and Tables

**Table 1.** Summary of considerations for deciding whether to include nonrandomized studies of interventions in systematic reviews of the effects of interventions

Systematic review step	Considerations

Topic scoping and refinement	Consider the following questions at a minimum when evaluating the potential utility of RCTs and NRSIs: 1. What are the decisional dilemmas and KQs being addressed, and how will the end-user(s) of the systematic review use the evidence to inform decision making? 2. Is it logical and likely for RCTs to have addressed the KQs adequately? 3. How serious is the risk of bias in NRSIs that address the KQs likely to be? 4. To what extent are NRSIs and RCTs likely to complement each other?
Systematic review team formation	When NRSIs are included: 5. Ensure that the systematic review team members are familiar with topic-specific data sources and advanced analytic methods for NRSIs.
Protocol development	The protocol should: 6. Specify study design methods or features eligible for the systematic review. 7. Explain whether and why NRSIs will be included or excluded. 8. Explain the potential implications of the decision to include or exclude NRSIs. 9. When NRSIs are included, describe the processes for synthesizing evidence from RCTs and NRSIs and determining overall strength of evidence.

*Abbreviations:* KQ, key question; NRSI, nonrandomized study of interventions; RCT, randomized controlled trial.

## What is new?

### Key finding

- We developed guidance consisting of nine considerations to inform decisions regarding the inclusion of nonrandomized studies of interventions (NRSIs) in systematic reviews (SRs) of interventions. The considerations were related to topic scoping and refinement (*n* = 4), SR team formation (*n* = 1), and protocol development (*n* = 4).

### What this adds to what was known?

- This guidance updates the 2010 guidance provided by the Agency for Health Care Research and Quality (AHRQ) Evidence-based Practice Center (EPC) Program that recommended NRSI inclusion only if RCTs are insufficient to address the key question.

### What is the implication and what should change now?

- This guidance may improve the applicability of an SR’s findings and their utility for end-users, but is likely to require more time, methodological expertise, and resources than would have been needed if NRSIs were excluded from the SR. Systematic reviewers should be transparent in justifying decisions regarding the inclusion of NRSIs in the SR protocol and the final publication.
